# Production of Enzymes from Agroindustrial Wastes by Biosurfactant-Producing Strains of *Bacillus subtilis*

**DOI:** 10.1155/2013/103960

**Published:** 2013-02-26

**Authors:** Francisco Fábio Cavalcante Barros, Ana Paula Resende Simiqueli, Cristiano José de Andrade, Gláucia Maria Pastore

**Affiliations:** Department of Food Science, Faculty of Food Engineering, University of Campinas, P.O. Box 6121, 13083-862 Campinas, SP, Brazil

## Abstract

Bacteria in the genus *Bacillus* are the source of several enzymes of current industrial interest. Hydrolases, such as amylases, proteases, and lipases, are the main enzymes consumed worldwide and have applications in a wide range of products and industrial processes. Fermentation processes by *Bacillus subtilis* using cassava wastewater as a substrate are reported in the technical literature; however, the same combination of microorganisms and this culture medium is limited or nonexistent. In this paper, the amylase, protease, and lipase production of ten *Bacillus subtilis* strains previously identified as biosurfactant producers in cassava wastewater was evaluated. The LB1a and LB5a strains were selected for analysis using a synthetic medium and cassava wastewater and were identified as good enzyme producers, especially of amylases and proteases. In addition, the enzymatic activity results indicate that cassava wastewater was better than the synthetic medium for the induction of these enzymes.

## 1. Introduction

The species of the genus *Bacillus* are known to be producers of enzymes of industrial interest. These bacteria are responsible for approximately 50% of the total enzyme market [1], which is estimated at 1.6 billion dollars. One of the primary advantages of using these species for enzyme production is that they are easily grown and maintained in the laboratory because they adapt to changes in the growing conditions that hinder the development and enzymatic synthesis of other microorganisms [2].

Among the different categories of enzymes, hydrolases are those with the largest industrial application, and among these, amylases, in particular alpha- and beta-amylases, have received special attention [3]. These enzymes catalyze the hydrolysis of starch and are produced by a wide range of microorganisms; however, for commercial applications, they are generally derived from *Bacillus* [1, 3–6], such as *B. licheniformis*, *B. stearothermophilus,* and *B. amyloliquefaciens*. These enzymes are applied to several industrial sectors, such as the food, fermentation, textiles, detergents, and paper industry [3, 5, 6]. The main amylases produced by *Bacillus* are resistant to heat, which is commercially important because numerous processes require high temperatures. Thus, the sensitivity to heat is no longer a limiting factor for their use [3].

Another relevant group is the proteases, which represent approximately 30% of the total sales of enzymes worldwide [7]. Proteases are predominantly applied to the food, textile, pharmaceutical, and detergent industries [1]. Some microorganisms produce low amounts of these enzymes, which impair their industrial application. However, in most cases, by adopting simple methods, such as the use of a specific and optimized medium, it is possible to increase production yields. The thermostable proteases produced by *Bacillus* spp. are among the most industrially important ones [7].

Finally, lipases, that is, enzymes that catalyze triacylglycerol hydrolysis, are widely used in organic chemistry due to their high specificity and selectivity [8]. Thus, they have received considerable attention because of their potential use in industrial processes [9], especially as biocatalysts. Among the reasons for the enormous potential of these enzymes are their high stability in organic solvents, the nonrequirement for cofactors, and broad substrate specificity [8]. However, these enzymes are only moderately stable at high temperatures where most industrial processes are performed. This drawback might be solved with the use of lipases produced by thermophilic microorganisms [8]. *Bacillus subtilis* secretes different lipases that vary according to growth and environmental conditions, pH, and amino acid supply [10].

The aim of this research project was to study the production of amylases, proteases, and lipases by *Bacillus subtilis* strains previously identified as biosurfactant producers, including the use of cassava wastewater as culture medium.

## 2. Materials and Methods

### 2.1. Microorganisms and Inoculum Preparation

The strains of *Bacillus subtilis* assessed in this study were ATCC 21332 from the *American Type Culture Collection* and LB2B, LB115, LB117, LB1a, LB5a, LB114, LB262, LB157, and LB2a from the culture collection of the Bioflavors Laboratory (DCA/FEA/Unicamp). All the strains were previously identified as producers of lipopeptide surfactants [11]. These cultures were maintained in inclined nutrient agar and refrigerated between 5 and 7°C.

For experiments in solid medium, the inoculum was prepared in Petri dishes containing nutrient agar (30°C, 24 h). The isolated colonies with specific growth characteristics (irregular shape, wavy edges, whitish, waxy, and flat top) were transferred using an inoculation needle to the solid media. In the liquid medium experiments the inoculum was produced as described by Barros et al. [12].

### 2.2. Culture Media

The culture media were classified into complex solids, synthetic liquids, and cassava wastewater. All media were maintained refrigerated until use.

#### 2.2.1. Complex Solid Media

The complex solid media used were the following.

- Differential agar for extracellular lipase-producing microorganisms as described by Lin et al. [13]. The composition percentages (w/v) in distilled water were olive oil 2.0, peptone 0.3, yeast extract 0.2, K_2_HPO_4_ 0.2, MgSO_4_·7H_2_O 0.1, Na_2_CO_3_ 0.1, agar 2.0, and rhodamine B 0.001.
- In order to test the production of extracellular protease, a medium was prepared as described by Giongo [14]. The concentration of each component (g·L^−1^) in distilled water was meat peptone 5.0, yeast extract 3.0, skimmed milk powder 10.0, and agar 12.0.
- To assess the presence of extracellular amylases, the medium described by Giongo [14] was adapted by the replacement of the skimmed milk powder with the same concentration of cassava starch.

#### 2.2.2. Synthetic Liquid Media

The liquid media were prepared from a basal medium with the following composition (g·L^−1^ of distilled water): yeast extract 1.0, KH_2_PO_4_ 2.0, (NH_4_)_2_SO_4_ 5.0, sodium citrate 1.0, MgSO_4_·7H_2_O 0.2, CaCl_2_·2H_2_O 0.01, FeCl_3_ 0.001, MnSO_4_·7H_2_O 0.001, and ZnSO_4_·7H_2_O 0.001. Supplements of carbon sources were added in a proportion of 1.0% (v/v) for liquids or (w/v) for solids according to the following description: olive oil for lipase, glucose for protease and cassava starch for amylase. After preparation, 50 mL of media was placed in 125 mL Erlenmeyer flasks and sterilized in an autoclave at 121°C for 20 min.

#### 2.2.3. Cassava Wastewater

Cassava wastewater from a cassava flour factory was heated until boiling, cooled until it reached 5°C, and centrifuged at 5°C and at 1,600 ×g for 20 min. This process was aimed at the solubilization of starch, the removal of suspended solids, and the elimination of hydrogen cyanide. After this process, the resulting liquid was called treated cassava wastewater. For the experiments, 50 mL of this medium was placed into 125 mL Erlenmeyer flasks and sterilized in an autoclave at 121°C for 20 min.

### 2.3. Culture Selection in Solid Medium

Each culture was inoculated using an inoculation needle in Petri dishes containing the specific medium for each experiment. All cultures were incubated at 30°C for 72 h, and the colony diameter and halo formation were each measured every 24 h. An average colony diameter was obtained by measuring two perpendicular axes. The halos were calculated as the total hydrolysis diameter minus the colony diameter. All experiments were performed in duplicate and repeated two times.

### 2.4. Fermentative Process in Liquid Medium

The sterilized treated cassava wastewater and the synthetic media were inoculated and incubated at 30°C with agitation at 150 rpm for 60 h. An amount of 1 mL of inoculum was added to each glass flask containing 50 mL of media. Samples were aseptically obtained at regular intervals to determine the enzymatic activity.

### 2.5. Fermentative Process in Bioreactor

Fermentations were carried out in bench bioreactor (3.0 L) with 1.5 L of sterile treated cassava wastewater, which was added to the bioreactor vessel 100 mL of the inoculum. The apparatus is best described by Barros et al. [12]. The operating conditions were temperature, aeration, and stirring maintained at 30°C, 1 vvm, and 150 rpm, respectively. Samples were aseptically obtained at regular intervals to determine the enzymatic activity. The enzymatic activities were also performed in recovered foam simultaneously times to the culture medium.

### 2.6. Enzymatic Activity Measurement

#### 2.6.1. Enzymatic Extract Preparation

Each sample was centrifuged at 3,900 ×g in an eppendorf centrifuge tube for 2 min to remove cells. The supernatant, called enzymatic extract, was used to determine the enzymatic activity.

#### 2.6.2. Proteolytic Activity Measurement

The proteolytic activity was based on the capacity of extracts to promote casein hydrolysis. To assess this activity, 0.5 mL of enzymatic extract was added to a casein solution 1.2% (w/v) in phosphate buffer 0.2 M (pH 7.0). After mixing vigorously, the flasks were incubated in a water bath at 37°C for 30 min. After incubation, the reaction was stopped by adding 4 mL of acetate buffer 0.2 M (pH 4.0), cooled in an ice bath (0°C) and filtered with filter paper. After filtration, 1 mL of the liquid was removed and added to 3 mL of NaOH 1 M and 0.5 mL of the Folin-Ciocalteau reagent diluted 1 : 1 of distilled water. Then, the absorbance of samples was measured in a spectrophotometer at 660 nm. A blank measurement was determined by adding distilled water instead of enzymatic extract for each group of samples. The enzymatic activity was determined using a tyrosine standard curve, and one activity unity (U) was defined as 1 *μ*moL of tyrosine released per mL of enzyme per hour [15].

#### 2.6.3. Amylolytic Activity Measurement

The amylolytic activity was based on the extract capability to promote starch hydrolysis. To assess this activity, 0.5 mL of extract was added to 5 mL soluble starch 1.0% (w/v) in phosphate buffer 0.2 M (pH 7.0). Flasks were incubated at 37°C for 10 minutes. After incubation, the reaction was stopped by adding 5 mL HCl 0.1 N. Then, 0.5 mL of this solution was added to 5 mL iodide-iodate 5–0.5% (w/v) and diluted 1 : 9 in phosphate buffer 0.2 M pH 7.0. In parallel, a blank sample and a substrate blank were used. The blank reaction was obtained in a similar manner as the samples, differing only by the addition of distilled water instead of extract. The substrate blank consisted of 5 mL HCl 0.1 N, 5 mL phosphate buffer 0.2 M (pH 7.0), and 0.5 mL distilled water. The absorbance of the samples was read in a spectrophotometer at 580 nm, and their activities were determined using a starch standard curve. One enzymatic activity unity was defined as the reduction of 0.1 mg of starch in 10 minutes of reaction with 0.5 mL extract [4].

#### 2.6.4. Lipolytic Activity Measurement

The lipolytic activity was based on the extract capability to promote the hydrolysis of olive oil triacylglycerols. In this experiment, 1 mL of extract was added to flasks containing 4 mL phosphate buffer 0.2 M (pH 9.0), 1 mL CaCl_2_ 110 mM, and 5 mL emulsion 25% (v/v) olive oil in Arabic gum 7% (w/v). The flasks were incubated at 37°C and 160 rpm for 20 min. After incubation, the reaction was stopped by adding 15 mL acetone : ethanol (1 : 1), and it was kept in a shaker for 5 more minutes. A blank sample was obtained in a similar manner as the extract samples, differing only by the use of distilled water instead of extract. The fatty acids released by the triacylglycerol hydrolysis were titrated with NaOH 0.05 M, and 20 *μ*L phenolphthalein solution (0.5% in ethanol (w/v)) was used as indicator. One lipase unity (U) was defined as the amount of enzyme able to release one *μ*mol of fatty acid per minute (*μ*moL·min^−1^) under the conditions described above [16, 17].

## 3. Results

### 3.1. Culture Selection in Solid Medium

**Figure 1:**
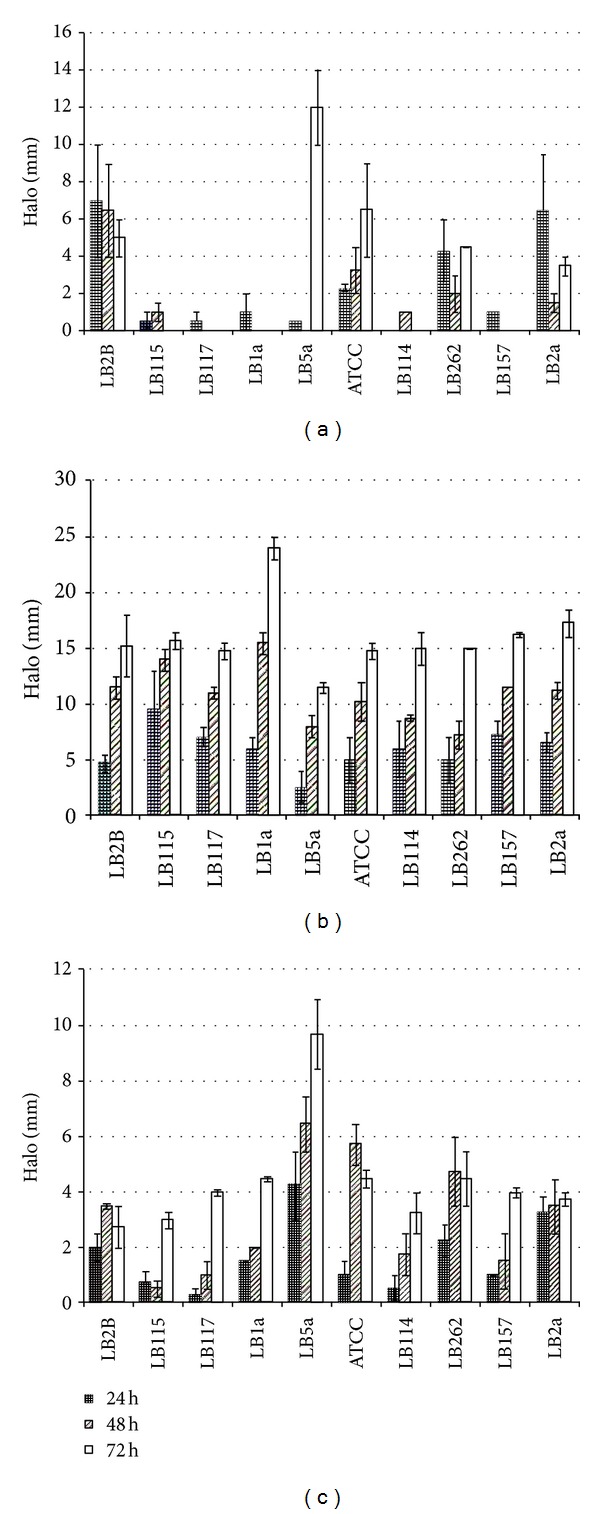
Average halo diameter in plates for LB1a and LB5a *Bacillus subtilis* strains: (a) amylase, (b) protease, and (c) lipase.

All strains showed a halo formation in all experiments for all enzymes (Figures 1(a), 1(b), and 1(c)).

**Figure 2:**
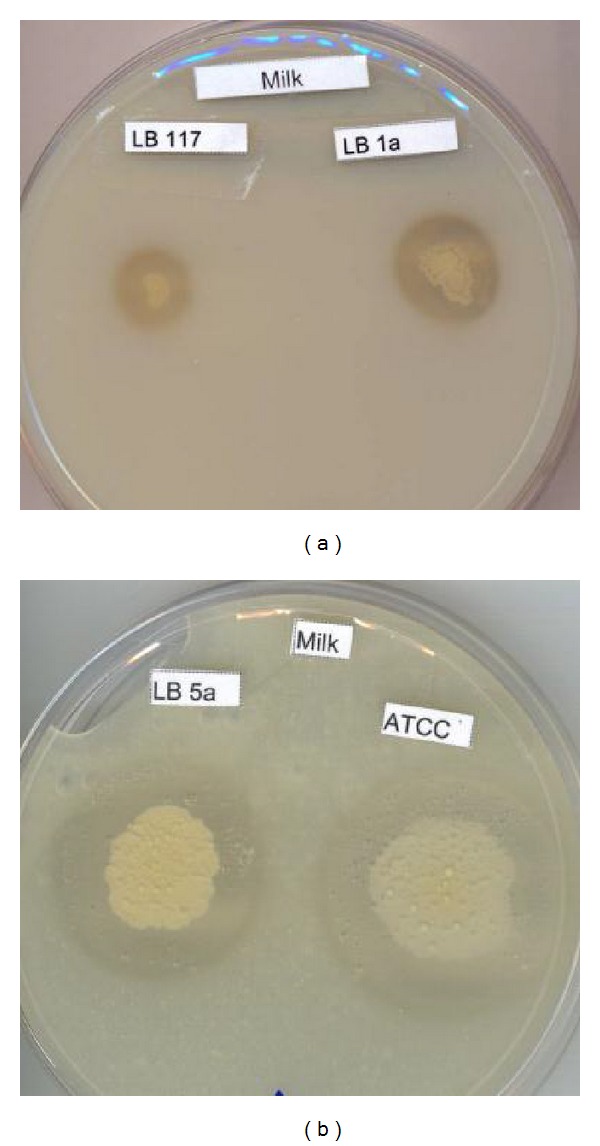
Milk agar plates after 24 h (a) and 72 h (b) inoculated with *Bacillus subtilis *strains LB117 and LB1a (a) and LB5a and ATCC21332 (b); the proteolysis halos are evident.

In the amylase production screening, the LB5a strain showed the largest halo at end of fermentation—even considering the considerable growth of the colony on the plate (12.0 mm). Its performance was substantially better than the second best strain, ATCC 21332, which showed an average halo diameter of 6.5 mm at 72 h (Figure 1(a)). For protease experiments (Figure 1(b)), the largest halos were produced by LB1a, which presented halo diameter of 24 mm, a significant vantage over the other strains at 72 h. All other strains showed values closer to 15 mm, except LB5a, which showed values slightly over 10 mm. For lipase, the largest halos were produced by LB5a strain, reaching a maximum halo diameter of 9.8 mm at 72 h. In some experiments there was a reduction in the halo size after 48 h (Figure 1(c)). The experiments are illustrated by Figure 2.

### 3.2. Enzymatic Activity in Synthetic Liquid Medium

**Figure 3:**
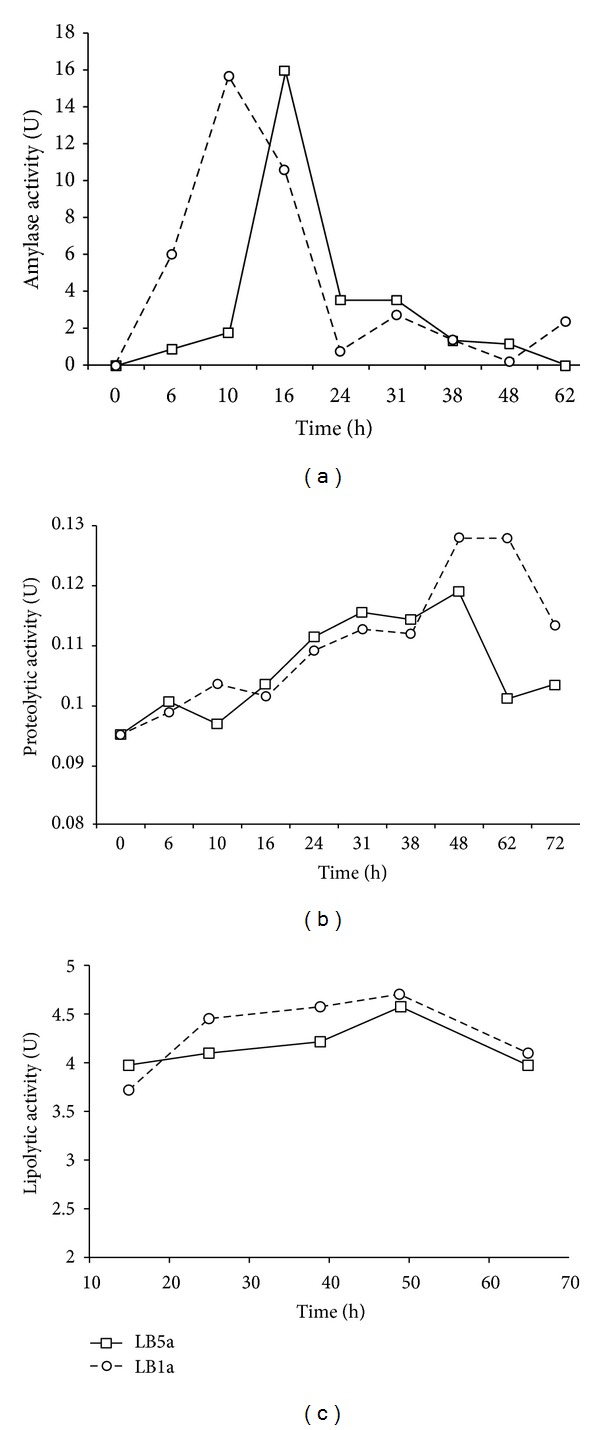
Enzymatic activity kinetics of LB5a and LB1a strains in a synthetic medium supplemented: (a) amylase, (b) protease, and (c) lipase.

All results of enzymatic activity in synthetic liquid medium are shown in Figure 3, which show enzymatic activity as function of fermentation time.

The amylolytic enzymes showed an intense activity increase at the beginning of the experiment. The maximum activity values were approximately 16 U for both strains, but the apex was reached at different times: 10 and 16 hours for LB1a and LB5a, respectively. After few hours, there was a strong reduction of activity to levels ranging from 4 to near zero U for all strains (Figure 3(a)). Concerning the protease activity, there was a gradual rise in their levels during almost all the fermentation processes, a behavior that was similar for both strains. The enzymatic activity reached its peak at values of approximately 1.2 U and 1.3 U, for LB5a and LB1a, respectively. The activity peaks were attained simultaneously at approximately 48 h. However, LB1a maintained values near the maximum until 62 h. After reaching the maximum, there was an accentuated reduction of activity to levels close to the fermentation beginning (Figure 3(b)). The lipolytic activity was very low through all processes, showing only small variations (Figure 3(c)).

### 3.3. Enzymatic Activity in Cassava Wastewater

**Figure 4:**
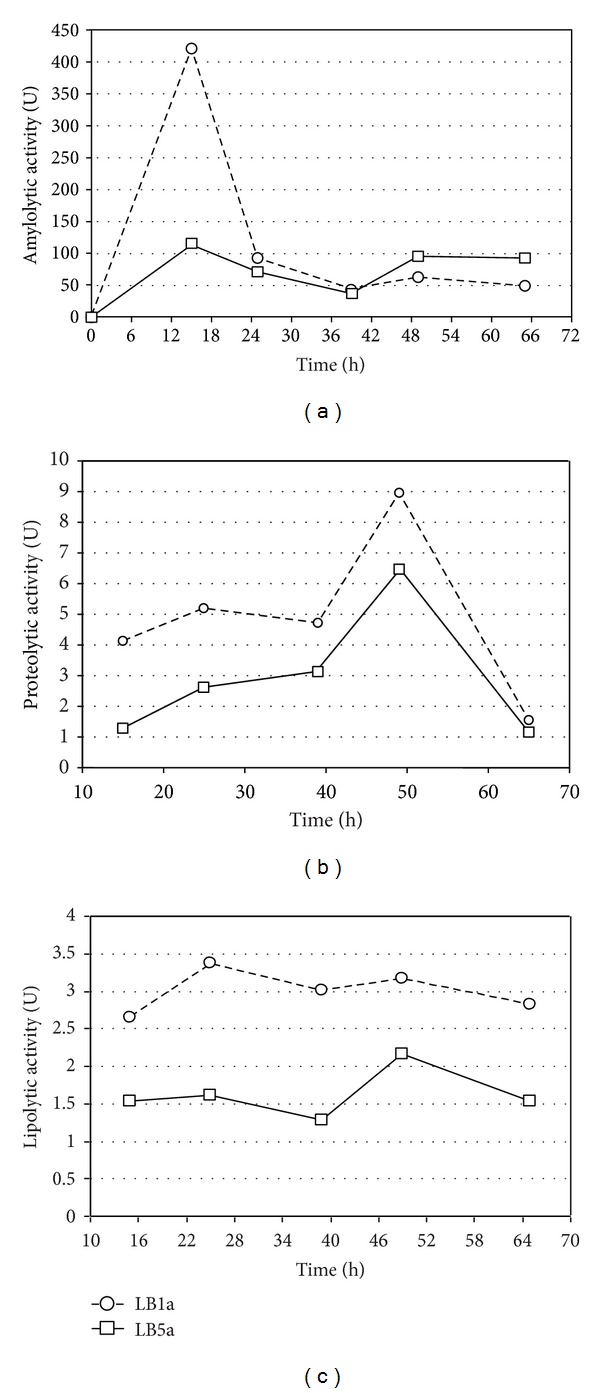
Enzymatic activity kinetics of LB5a and LB1a strains grown in glass flasks using cassava wastewater as culture medium: (a) amylase, (b) protease, and (c) lipase.

All results of enzymatic activity in cassava wastewater medium are shown in Figure 4, which shows enzymatic activity as function of fermentation time.

For amylases the results were substantially high when compared with results reached in synthetic culture medium supplemented with soluble starch. In cassava wastewater medium, while LB1a reached maximum over 400 U, LB5a reached about 150 U. Regarding the kinetic behavior, both strains were similar to each other and when compared to the results obtained in the synthetic medium they showed a significant increase of activity in the first hours followed by a considerable reduction after a short time (Figure 4(a)).

The proteolytic activities achieved by both strains were higher throughout the process in cassava waste than in synthetic medium (Figure 4(b)). However, the maximum values for both strains were reached at similar periods to those found in the synthetic medium, that is, at approximately 50 h. Such as in the synthetic medium, LB1a also showed the highest peak in the wastewater. Comparing the curves of both strains (Figure 4(b)), it is possible to see consistently higher results for LB1a than those found with LB5a during the whole process. Only close to the end of the fermentation process, after the peak was reached, the difference between the culture media became insignificant, especially due to the considerable reduction in the activity in both media.

The lipolytic activity values remained nearly constant for both strains throughout the studied period (Figure 4(c)). However, the activity in cassava wastewater was always lower than in the synthetic medium during the whole fermentation process for both microbial cultures. The reduction was more intense for LB5a, which showed approximately 50% decreased activity in the cassava wastewater when compared with the synthetic medium. In contrast, the reduction for LB1a oscillated at about 30%.

### 3.4. Enzymatic Activity in Bench Bioreactor

**Figure 5:**
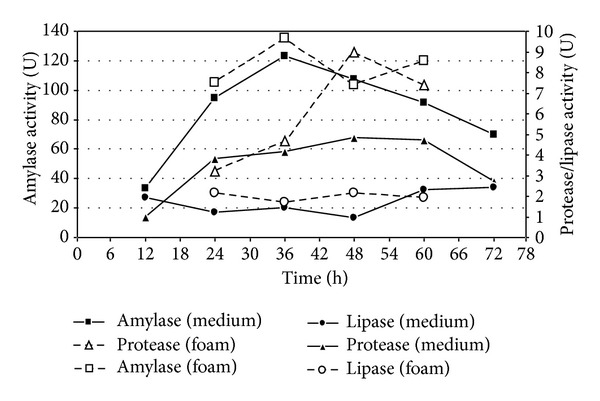
Enzymatic activity kinetics of LB5a and LB1a strains grown in bioreactor using cassava wastewater as culture medium: (a) amylase, (b) protease, and (c) lipase.

As seen in Figure 5, there is a strong increase in amylase activity in the culture medium at the first hours, from near to 30 U at 12 h to values around 120 U at 36 h. After that, the activity was gradually reduced until the end of fermentation. The measurement of amylolytic enzymes in the foam recovered from bioreactor did not show significant difference between the values found in foam and culture medium. For protease, the curves started from 10 U at the fermentation initiation to values near 65 U between 48 and 60 h. After this maximum period, the value dropped to 35 U in 12 h. It was possible to observe after 36 h a significant difference between the enzymatic activity in the foam and the culture medium, where the values found in foam was always higher. For lipase, the values were very low since beginning and continued very low until the end of fermentation. There was no significant difference between foam and culture medium.

## 4. Discussion

### 4.1. Culture Selection in Solid Medium

The halo formation in all experiments indicated that all tested strains produce extracellular enzymes which were able to hydrolyze their respective substrates.

In general way, in amylase experiment the colonies displayed substantial development, which was higher than that found in the lipase and protease tests, thereby it was not possible to measure the halo of all strains at all times. The high speed of growth of the colonies could be explained by the fact that carbohydrates are the preferred carbon source for microorganisms of the genus *Bacillus* [12, 18]. Likewise, considering that the ultimate goal was the production of enzymes in cassava wastewater, the results of starch hydrolysis of the LB5a strain represented a critical finding. Considering the best results at 72 h, LB5a strain was selected to the following step.

The smallest protease halo shown by LB5a was opposite to the behavior observed in the amylase and lipase activity experiments (Figure 1). In the other hand, the LB1a strain, which had poor amylolytic and moderate lipolytic activities, showed the largest halo formation in the protease activity test. Considering the best results at 72 h, LB1a strain was selected for the activity experiments in liquid medium.

For lipase, the LB5a strain showed the best results in all the measurements (Figure 1(c)), which allow us to infer a good activity. This is in accordance with previous works that relate the use of solid media supplemented to emulsified triglycerides as standard methodology for the selection of lipase-producing microorganisms [17, 19]. In the other hand, although there was no strong positive relationship between the size of a halo in solid medium and the production of alkaline lipase in liquid medium, there is a greater tendency for microorganisms that showed a bigger halo to have a greater lipolytic activity in liquid medium [17]. Thus, the LB5a strain was selected for additional experiments. It is important to observe that the reduction of halo diameter after 48 h in some strains (Figure 1(c)) is related to faster growth of colonies rather than the formation of halos.

In addition, both selected strains revealed good biosurfactant production, in which the production from LB5a was higher than LB1a, as reported by Nitschke et al. [11]. The enzyme production coupled with biosurfactant could make these strains economically interesting, since they could be suitable for an integrated production process.

### 4.2. Enzymatic Activity in Synthetic Liquid Medium

All experiments aiming to detect enzymatic activity in synthetic liquid medium confirmed the data found in the solid medium screening. The presence of enzymatic activity for the two strains previously selected was confirmed.

The intense increase of amylolytic activities was similar to LB1a and LB5a (Figure 2(a)). These demonstrate that the studied bacteria were capable of using cassava starch as carbon source since it was the only C source available in the medium. Moreover, the production of enzymes occurred in the first hours of fermentation as expected. However, the different times found to the apex, 10 and 16 hours for LB1a and LB5a, respectively, showed that two strains present different abilities in the use of this substratum. Although the process happened more quickly in this experiment in comparison with other studies, the intense increase in activity at the start of the fermentation process is consistent with the data found in the collected works [6]. In this study the peak of enzymatic production ranged between 20 and 36 h, depending on the conditions used. The intense reduction of activity just after maximum could be interrelated to the depletion of the starch in the culture medium.

There is an apparent discrepancy of these results when compared with those found in solid medium experiments (Figure 1(a)) since LB1a growth produced only small amylase halos at 24 h, while no halos were visualized at 48 and 72 h. However, these data may not indicate a low enzymatic activity. This supposition is corroborated by the considerable culture growth on plates with starch-rich culture medium; thus, the colony spread faster than the hydrolysis halo. In other words, this evidence indicates that the cell development was strong because this medium provided better conditions to the microorganisms' survival and growth. This outcome demonstrates that both strains were highly adapted to the use of cassava starch as a carbon source.

Although the protease activity in solid medium was consistently higher for the LB1a, in liquid medium only at maximum activity there was a significant advantage of this strain. Nevertheless, the activity can be considered low. The reduced activity throughout the process to both strains (Figure 2(b)) might be related to the fact that the cassava wastewater is not necessarily an inductor since the C source used was glucose and there was no protein substrate. Moreover, the strains were previously selected as good biosurfactant producers [11], and as the degradation of the biosurfactant produced by *Bacillus subtilis* 21332 at late fermentation time may have a direct liaison with the protease activity of the medium [18]; thus, the low activities found were probably a condition for their high biosurfactant production. This supposition is supported by the maintenance of the maximum activity for long periods at the end of fermentation, which is also related to the results obtained using the solid medium (Figure 1(b)). Finally, the accentuate decrease near the end of fermentation might be related to the total depletion of carbon sources and the beginning of the death phase of the cellular growth.

Very low levels of lipase activity even in the presence of inducer show that the lines are not good producer of this enzyme.

### 4.3. Enzymatic Activity in Cassava Wastewater

The production of biosurfactants by *Bacillus subtilis* in cassava wastewater was reported by Nitschke et al. [11], Barros et al. [12], and Barros et al. [20]. However, the use of the combination of these microorganisms and substrate for the enzyme production was not previously described. Thus, to evaluate this potential, the same strains were used in synthetic medium essays—LB1a and LB5a. As seen in all experiments, both strains were capable of producing extracellular enzymes in cassava wastewater. However, the use of a complex medium instead of a synthetic one affected the results significantly.

The amylose activity of cultures grown in cassava wastewater was higher than at in the synthetic medium (Figure 3(a)). The difference also was found when comparing the activity levels of the two strains. Hence, unlike the synthetic medium, in which similar values were found for both microorganisms, in cassava wastewater the LB1a strain showed values approximately 40 times higher than in the synthetic medium, while the LB5a values were only 10 times higher. The difference between peaks of enzymatic activity might be related to the results found in the synthetic media. For instance, LB5a showed short period at high level of activity in synthetic medium while LB1a presented a longer time at high levels (Figure 2(a)). Besides, in synthetic medium, the LB1a reached high activity levels before LB5a.

Cassava wastewater has a combination of sugars of high molecular weight, mainly soluble starch, and low molecular weight sugars, primarily saccharose, fructose, and glucose. The latter were preferentially consumed by the cultures. Consequently, after the depletion of the lower molecular weight sugars, the cultures started to produce amylases to provide glucose for their metabolism. One indication of this behavior was shown at previous experiments [12], which were was performed fermentations with the LB5a strain in cassava wastewater using a bioreactor. They detected a rise in reducing sugars concentration in the first 12 h of the fermentation, while the total sugars concentration had a sharp decline. The elevation of low molecular weight sugars is from the hydrolysis of starch.

The higher production of protease when compared with synthetic media could be explained by the waste composition, since it is very rich in important nutrients for *Bacillus* development [12]. The behavior of kinetics is very similar to that found in this work for synthetic medium.

Finally, the low lipolytic activity in both strains might be explained by the absence of an inductor in cassava wastewater beyond the facts already discussed earlier.

### 4.4. Enzymatic Activity in Bioreactor

The kinetic curves of amylase activity in bioreactor are consistent with literature data [4], where the peak of enzyme production is around 24 and 36 hours according to the variation in the conditions used, although the composition of the medium is different. The initial increase in amylase activity is obviously due to the presence of starch in the composition of the culture medium. Subsequently, starch hydrolysis released glucose to the microbial culture. Thus, this increase is not completely simultaneous with the exponential growth phase. Cell growth in the early hours of the fermentation is supported by the low molecular weight sugars like glucose, fructose, and sucrose. The effective use of glucose from starch hydrolysis by the microbial culture occured only after depletion of culture medium glucose content. Similar results are presented in cassava by Nitschke [18] and Barros et al. [12].

The enzyme activity also was measured in the foam removed from the fermenter. This aspect was considered important, since in this case, the primary recovery system of the biosurfactant is based on the principle of the bubble column, a method used for recovery and purification of surfactants [21] and enzymes [16]. Thus, different activities were expected between the activities found in the middle and in the foam. However, considering the activity found in the foam, despite having higher activities in most of sampling time (except for the last measurement); there, were no significant differences between the values found in the fermented medium. Thus, recovery was not detected for these categories of enzymes, because this recovery could be easily evidenced by a higher activity in the foam, which was not the case. In fact, there is no a similar behavior for all proteins recovery in the recovery using bubble column [21]. 

As in experiments in glass flasks, the increase of proteolytic activity was gradual, reaching a maximum between 48 and 60 hours. The low activity even at its apex may be related to the absence of protein that could be an inducer. Furthermore, as discussed before, the LB5a was previously selected as good biosurfactant-producing strain, which could be related to a low proteolytic activity. Although their values are increasing since the early hours, the proteolytic activities do not appear to be directly linked to microbial growth, because their maximum values are reached only about 24 hours after the end of exponential growth phase.

The proteases apparently are recovered by foam. In this case, the enzyme activities were significantly higher in the foam, especially at peak times, with values about twice greater in the foam than in the culture medium. Despite being significant, this enhancement factor was smaller than that for the surfactin, whose concentrations in the foam can reach up to 60 times the concentration in the culture broth. Regarding the lipase, as the flask experiments, the activity was low at all times, both in the culture medium and in the foam. There was no significant difference between the values of activity found in both.

## 5. Conclusion

Among the 10 strains of* Bacillus subtilis* tested for the production of enzymes, LB1a and LB5a were identified as potential sources of amylases and proteases. The production of these two enzymes in cassava wastewater was better than that in the synthetic medium, showing the great potential of this agroindustrial residue for use as an alternative substrate. Similarly, the use of cassava wastewater as a substrate for enzyme production by *Bacillus* has not been reported in the literature until now. Additionally, the protease produced by the strain was carried by the foam produced during fermentation, which provides a simpler and economically feasible recovery. 

Our results indicate the possibility of an integrated process for obtaining these enzymes and biosurfactants, which were also produced by these strains during the fermentation of cassava residue, that could greatly increase the economic viability of these strains.
